# How to think like an emergency care provider: a conceptual mental model for decision making in emergency care

**DOI:** 10.1186/s12245-020-00274-0

**Published:** 2020-04-16

**Authors:** Nasser Hammad Al-Azri

**Affiliations:** Emergency Department, Ibri Hospital, Ministry of Health, POB 134, 516 Akhdar, Ibri, Oman

**Keywords:** Decision-making, Emergency care, Emergency medicine, Mental model, Situational model

## Abstract

**Background:**

General medicine commonly adopts a strategy based on the analytic approach utilizing the hypothetico-deductive method. Medical emergency care and education have been following similarly the same approach. However, the unique milieu and task complexity in emergency care settings pose a challenge to the analytic approach, particularly when confronted with a critically ill patient who requires immediate action. Despite having discussions in the literature addressing the unique characteristics of medical emergency care settings, there has been hardly any alternative structured mental model proposed to overcome those challenges.

**Methods:**

This paper attempts to address a conceptual mental model for emergency care that combines both analytic as well as non-analytic methods in decision making.

**Results:**

The proposed model is organized in an alphabetical mnemonic, A–H. The proposed model includes eight steps for approaching emergency cases, viz., awareness, basic supportive measures, control of potential threats, diagnostics, emergency care, follow-up, groups of particular interest, and highlights. These steps might be utilized to organize and prioritize the management of emergency patients.

**Discussion:**

Metacognition is very important to develop practicable mental models in practice. The proposed model is flexible and takes into consideration the dynamicity of emergency cases. It also combines both analytic and non-analytic skills in medical education and practice.

**Conclusion:**

Combining various clinical reasoning provides better opportunity, particularly for trainees and novices, to develop their experience and learn new skills. This mental model could be also of help for seasoned practitioners in their teaching, audits, and review of emergency cases.

## Background

“It is one thing to practice medicine in an emergency department; it is quite another to practice emergency medicine. The effective practice of emergency medicine requires an approach, a way of thinking that differs from other medical specialties” [1]. Yet, common teaching trains future emergency practitioners to “practice medicine in an emergency department.”

Emergency care is a complex activity. Emergency practitioners are like circus performers who have to “spin stacks of plates, one on top of another, of all different shapes and weights” [2]. This can be further complicated by simultaneous demands from various and multiple stakeholders such as administrators, patients, and colleagues. Add to that the time-bound interventions and parallel tasks required and it can be thought of no less than being chaotic.

There is a tendency to distinguish emergency care from other medical practices as being more action-driven than thought-oriented [3]. This probably stems from the presumption that emergency medicine follows the same strategy as other medical disciplines so it is judged within the same parameters. Another explanation for this is that emergency practitioners are seen to act immediately on their patients when other medical specialties might take longer time preparing for this action. However, the chaotic environment is different and it requires complex decision-making skills and strategies. Unlike general medical settings, in EM, often a history is unobtainable, and a physical examination and medical investigations are not readily available in a critically ill patient. Despite this, emergency medicine is still being taught using the conceptual model of general medicine that follows an information-gathering approach seeking optimal decision-making. In medical decision-making, the commonly adopted hypothetico-deductive method involving history taking, physical examination, and investigations corresponds to the general approach of medicine.

## Methods

### Importance of rethinking existing medical emergency care mental model

Education in medical emergency care adopts a strategy similar to that of general medicine despite the fact that it is not optimal in emergency departments. Emergency care providers cannot anticipate what condition their patients will be in and they cannot follow the steps of detailed history taking, complete physical examination, ordering required investigations, and, using the results, plan the management of their patient. Classical clinical decision theory may not fit dynamic environments like emergency care. Patients in the emergency department are usually critical, time is limited, and information is scarce or even absent, and decisions are still urgently required.

**Fig. 1:**
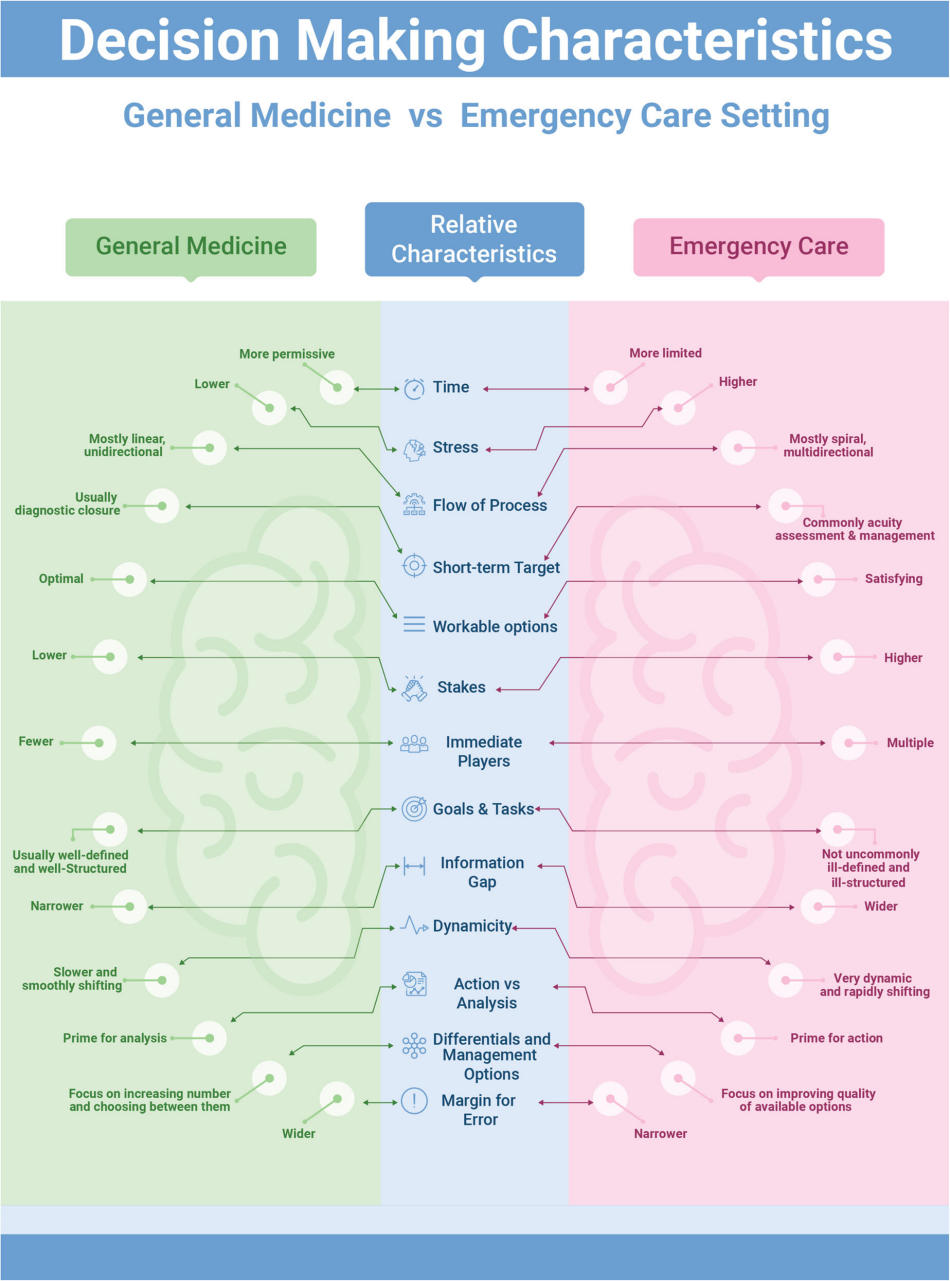
Comparing conventional decision-making in general medicine vs. emergency care setting

Croskerry (2002) has noted: “In few other workplace settings, and in no other area of medicine, is decision density as high” [4] as in emergency medicine. In an area where an information gap can be found in one third of emergency department visits, and more so in critical cases [5], an information-seeking strategy is unlikely to succeed. Moreover, diagnostic closure is usually the short-term target in the hypothetico-deductive method while this is less of a concern in emergency care. Instead, the short-term priorities in emergency care include assessment of acuity and life-saving [6]. Figure 1 presents a comparison of the conventional general medicine decision-making approach and how emergency care setting differs relatively with regard to those basic characteristics.

Hence, a different mental model with a distinctive approach for emergency care is required. Mental models are important to describe, explain, and predict situations [7]. This is the roadmap through the wilderness of emergency care rather than a guide on driving techniques. Experts are differentiated from novices in several aspects: sorting and categorizing problems, using different reasoning processes, developing mental models, and organizing content knowledge better [8]. In addition, experienced physicians form more rapid, higher quality working hypotheses and plans of management than novices do. Novices are especially challenged in this area, since teaching general problem solving was replaced with problem-based learning, as the emphasis shifted toward “helping students acquire a functional organization of content with clinically usable schemas” [9]. The proposed model is intended to better organize the knowledge and approach required in emergency care, which may eventually help improve the practice, particularly of novices.

Clinical decision-making in emergency care requires a unique approach that is sensitive to the distinctive milieu where emergency care takes place [10]. Xiao et al. (1996) have identified four components of task complexity in emergency medical care [11]. These include multiple and concurrent tasks, uncertainty, changing plans of management, and compressed work procedures with high workload. Such complex components require an approach that accommodates such factors and balances the various needs in a timely and priority-based, situationally adaptable methodology.

## Results

### A different model for emergency care

**Fig. 2:**
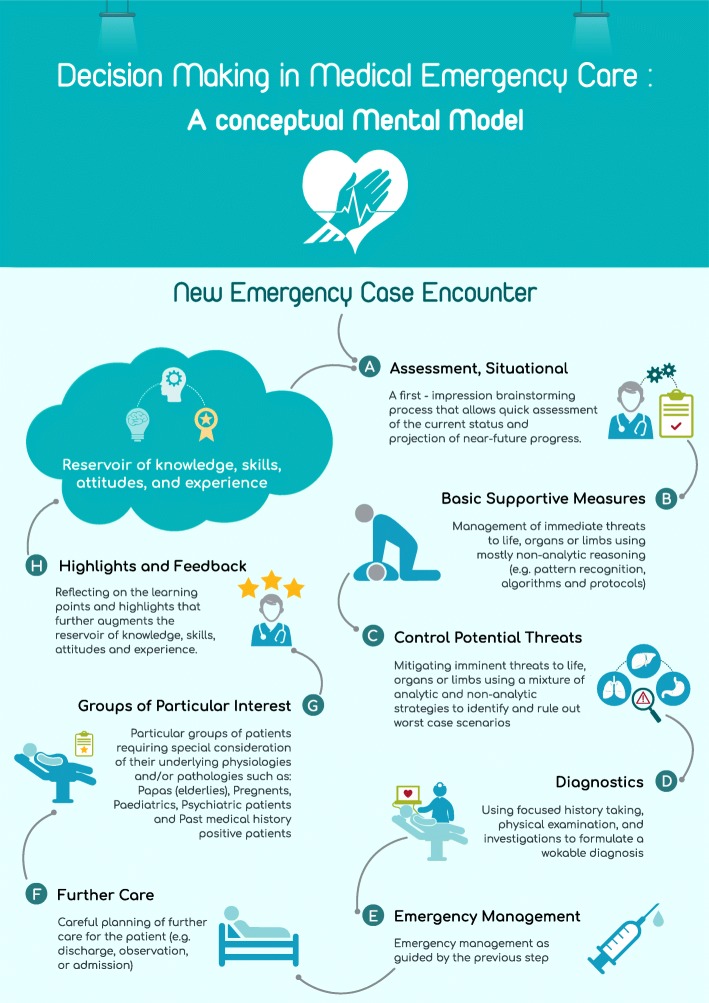
Situational decision-making model lifecycle

This article addresses a general mental approach involving eight steps arranged with an initialism mnemonic, A–H. Figure 2 presents an infographic of the lifecycle of this A–H decision-making process. These steps represent the lifecycle of decision-making in emergency practice and form the core of the proposed conceptual model. Every emergency care encounter starts with the first step of situational awareness (A) where the provider starts to build up a workable mental template of the case presentation. This process is ongoing throughout the encounter to reflect the dynamic nature of emergency cases. The second to fourth steps (B–D) involve a triaging process in order to prioritize the most appropriate management at that point in time, through a series of risk-stratification stages. Then, additional emergency management (E) follows based on the flow of the case from earlier steps. Following emergency management, a planning step regarding further care (F) for the patient is required. The following step concerns emergency patients who may represent special high risk groups (G) with special precautions and particular diagnostic and management approaches to be considered. This step is, in fact, a mandate throughout the process but included here as a reminder. The final step is a reflection of the entire process that highlights (H) the learning aspects from the case management. Throughout the process, the first and last steps are ongoing as they reflect the dynamicity of the situation.

#### A: (awareness, situational)

It is likely that the first thought of an emergency care provider, when confronted with an acutely ill patient, is the issue of time: “how much time do I have to act and how much time do I have to think?” [12]. The mental brainstorming that takes place in a matter of seconds is a very valuable and indispensable part of every single emergency encounter. Providers’ prior beliefs, expectations, emotions, knowledge, skills, and experience all contribute to the initial approach adopted. Individuals vary in the importance they attach to different factors [13], and this variation is reflected in the decisions they make. The importance of this mental process is, unfortunately, not reflected in either general medicine or emergency medicine education and research. Traditionally, “medical education has focused on the content rather than the process of clinical decision making” [6].

The notion of “situational awareness” (SA) is a useful concept to borrow from aviation sciences. Situational awareness has been defined as the individual’s “perception of the elements of the environment within a volume of time and space, the comprehension of their meaning and the projection of their status in the near future” [14]. As noted from the definition, SA tries to amalgamate the experiences and background of the practitioner with the current situation in order to enable a more educated prediction of what will happen next. Although the concept originated outside of the medical field, it has already been utilized in several medical disciplines including surgery, anesthesiology, as well as quality care, and patient safety [15–17]. Moreover, SA has been discussed in several emergency care mandates and it is recommended for inclusion in the non-technical skills training of teams in acute medicine [15].

This emphasizes that an attentiveness to the dynamic nature of priorities in emergency management is as important as knowledge and skills. As such, SA provides a mental model that encourages emergency care practitioners to stay alert for changes in the surrounding environment and relate those changes to case management. The importance of this step in the model is that it prods us to go beyond our immediate perceptions and gut feelings and develop an overall view of the situation [18]. Practically, decision-making in emergency care has historically depended more on rapid situational assessment rather than optimal decision-making strategies as in the hypothetico-deductive method [19]. SA is probably one of the most neglected, yet distinguishing, skills in emergency medicine education.

#### B: (basic life, organ, and limb supportive measures)

The second step in emergency decision-making involves a clinical triaging process. The purpose of this triage is to prioritize time-bound interventions or treatment for the patient. Immediate risks to life, organs, or limbs take priority in case management. This precedes any analytical thinking provided by detailed history taking, physical examination, or investigations, even though a focused approach might be necessary. This step maintains the dynamicity of the process of decision-making and allows the practitioner a holistic view of available and appropriate options rather than ordinary linear thinking. It also provides flexibility of movement between treatment options in response to dynamic changes in the condition.

Life-threatening conditions always take precedence in emergency management. The next priority is to manage immediate risks to body organs or limbs; this is the essence of medical emergency management. Therefore, the aim of this step on basic supportive action (B) is to save the vitals of the patient. This is where advanced cardiac and trauma life support algorithms and emergency management protocols are important.

A useful approach at this step is pattern recognition. In real practice, when confronted with a critically ill or crashing patient, the emergency care provider usually abandons the time-consuming hypothetico-deductive method; pattern recognition offers a rapid assessment and clinical plan that permits immediate life-, organ-, or limb-saving measures to take place [20]. Pattern recognition, known also as non-analytic reasoning, is a central feature of the expert medical practitioner’s ability to rapidly diagnose and respond appropriately, compared to novices who struggle with linear thinking skills [21–23]. This approach could be further augmented by the availability of algorithms and protocols that allow immediacy of perception and initiation of management [4], as well as by including it in clinical teaching and education.

#### C: (control potential life, organ, and limb threats)

While emergency care providers must prioritize immediate threats to life, organs, and limbs, they must also anticipate and recognize imminent threats to the same and control them (C). This is one of the biggest challenges in emergency care compared to other medical settings; oftentimes, the grey cases are the hidden tigers. In fact, seasoned emergency care providers know that even the most unremarkable patients may have a catastrophic outcome within moments [24]. Emergency care providers usually adopt mental templates for the top diagnoses that they need to exclude for every particular presentation. This is a step of “ruling out” worst diagnoses before proceeding. Croskerry (2002) asserts that this “rule out the worst case” strategy is almost pathognomonic of decision-making in the emergency department [4]. Many emergency presentations (e.g., poisoning, head injury, and chest pain) are true time bombs that any emergency care provider should be alert to.

This step presents an intermediate stage between the previous step (B) where pattern recognition and non-analytic reasoning dominates decision-making, and the next step (D) where the hypothetico-deductive approach with its analytic reasoning starts to play a major role in decision-making. As such, this step utilizes a mixture of the analytic and non-analytic reasoning to aid emergency care practitioners the “rule out the worst case” scenario in their patients. Examples of presentation-wise “worst case” scenarios are illustrated in Table 1.

**Table 1:** Examples of presentation-wise “worst case” scenarios

Presentation	Examples of worst case scenarios
Abdominal pain	• Abdominal aortic aneurysm • Mesenteric ischemia • Acute myocardial infarction • Perforated ulcer • Volvulus • Intussusception • Ovarian torsion • Ectopic pregnancy • Bowel Obstruction • Acute appendicitis • Cholangitis • Splenic sequestration in sickle cell Disease patients • Diabetic ketoacidosis • Black widow spider bite
Chest pain	• Pneumothorax/pneumomediastinum • Pericardial effusion/tamponade • Acute coronary syndromes • Pulmonary embolism • Acute aortic dissection • Traumatic aortic rupture • Pneumonia • Esophageal rupture • Acute chest syndrome in sickle cell Disease patients • Pericarditis
Eye pain and redness	• Acute angle-closure glaucoma • Orbital cellulitis • Anterior uveitis/iritis • Ruptured globe • Corneal abrasions/ulcer • Keratitis • Chemical burns
Headache	• Meningitis • Subarachnoid hemorrhage • Carbon monoxide poisoning • Intracranial abscess • Hematoma subdural/epidural • Temporal arteritis • Complicated sinusitis • Cavernous sinus thrombosis • Brain tumor • Acute angle-closure glaucoma

Once a potential threat is discovered, the practitioner will be situationally more aware and this will help to initiate measures that could prevent further deterioration of the condition. Again, this step is another that is practiced commonly by expert practitioners but is presented informally or insufficiently in emergency medicine training or education. Emergency care practitioners should focus more on this step due to its centrality in emergency care practice as well as its importance for ensuring safety of patients.

#### D: (diagnostics)

Once immediate and/ or imminent threats have either been excluded or managed, the emergency care provider may move on to the next step of formulating a workable clinical diagnosis (D) through the commonly adopted hypothetico-deductive medical model via a focused history taking, physical examination, and investigations. This is basically what all medical students are trained for in their undergraduate and postgraduate medical education. This step involves the utilization of existing tools for optimal decision-making within the available resources in the emergency department. Nevertheless, a final diagnosis may not be reachable in the emergency department setting.

#### E: (emergency management)

This is the step that naturally follows the diagnostic step (D). After collecting appropriate information regarding patient presentation through a focused history, examination and investigations, the emergency care provider may start emergency management and treatment as indicated. This does not contradict utilizing appropriate interventions in earlier steps (B, C) that aim to save life, organs, or limbs.

#### F: (further care)

While decisions about intervention(s) in emergency care are very difficult, often decisions about the further management of the patient are just as difficult [25]. Grey cases present the dilemma of whether to admit, keep for observation, or discharge. This decision is problematic because it entails not only technical aspects of the clinical status of the patient but also social, political, economic, and administrative factors along with the availability of supportive resources.

The initial brainstorm regarding imminent threats to life, organs, and limbs (C) continues to play a major role in the emergency provider’s decision-making. Discharging patients to their home carries risks related to a lack of clinical care and formal monitoring compared to admitted patients [26]. Hence, this step is pivotal in the emergency care of patients with significant implications in terms of outcome. Incorporating this step in the model is essential for the emergency care provider to have an integrative and holistic view of the case.

#### G: (groups of particular interest)

Certain groups of patients warrant particular concern while being managed in emergency care settings [27]. There are different reasons to consider these groups as high risk. Often, it is because they have underlying pathologies and/or physiologies that make them more prone for complications, acute exacerbations, and/or they are less likely to withstand the stress of acute illness. These groups include the elderly, pregnant women, children, psychiatric patients, and patients with a significant past medical history. These patients should cause particular concern that may justify a different and/or altered path of management at any step during the emergency care process.

#### H: (highlights)

Lack of informative feedback is one of the major drawbacks in emergency medicine that hinders learning and maintaining of cognitive and practical emergency care skills [28]. Feedback and highlighting of learning points is a crucial step in medical education and can be done in a variety of methods [29]. This is an ongoing step that starts at the case encounter and never ends during a practitioner’s career. Here, the practitioner reflects on the care and management provided during the encounter and makes a case for learning and advancing his knowledge, skills, and attitudes in emergency care. This step is usually done unconsciously. However, exposing this process to scrutiny and making it a formal step in the process of emergency care is likely to enhance experiential learning of the provider and, more importantly, offer feedback for the first step in the model that further augments situational awareness (A). This will add to the reservoir of understanding and attentiveness for future cases.

## Discussion

Thinking about thinking, also called metacognition, in emergency care is likely to reveal the strengths and weaknesses in current approaches and open doors for further development and improvement of emergency care. It is also likely to aid in recognizing opportunities for interventional thinking strategies [18]. This could be a step forward in preparing a broad-based, critical thinking pattern for physicians, who may save lives, organs, and limbs based on undifferentiated cases without having to depend on a diagnosis to do so.

The presented conceptual model attempts to contribute to the exposition and development of the forgotten skill of clinical reasoning with a particular reference to emergency and acute care. Moreover, it dissects the usually overlooked process of decision-making in emergency care [28]. The arrangement of the model components in alphabetical mnemonics may act as a reminder of a decision process that will reduce omission errors in clinical settings. Furthermore, functional categorization of the steps involved in decision-making, as well as in actual practice, will provide and develop further insight and awareness of cognitive strengths and weaknesses at different stages.

**Fig. 3:**
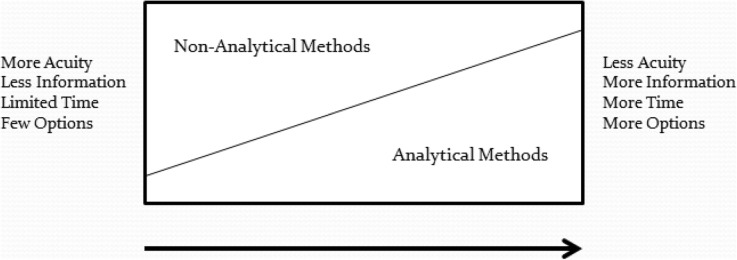
Situationally combined analytical and non-analytical decision-making methods

A significant advantage of the proposed conceptual mental model for emergency care is that it combines both analytic as well as non-analytic (also called naturalistic decision-making, NDM) strategies to aid medical emergency management. This model does not eliminate the need for the hypothetico-deductive analytic method but rather incorporates it within a more comprehensive approach and utilizes it when it is situationally appropriate along with the non-analytic method (Fig. 3). Combining different clinical reasoning strategies helps novice practitioners have greater diagnostic accuracy, improve performance, and avoid giving misleading information [30, 31].

In addition, emergency care has been described as chaotic. Chaotic contexts are characterized by dominance of the unknowables, indeterminate relationships between the cause and effect, and a lack of existing manageable patterns [32]. In such contexts, the best approach to management is to act to establish order, then sense where stability is present and where it is not, and then respond to transform the situation from chaos to complexity [32]. The described model addresses those activities in order where the emergency care provider first acts (B), then senses (C), and finally responds (D, E) to establish a more stable context.

The suggested approach can be utilized by various groups of practitioners, such as physicians, nurses, and paramedics, hence the use of the term emergency care. Moreover, novices and trainees learn better by being exposed to the decision-making process involved, rather than just mimicking the actions of experts [3].

## Conclusion

Medical education is required to produce a “broad-based physician, geared to solving undifferentiated clinical problems” [33]. Emergency medicine, as a generalist discipline, has probably high potential for that. The presented model could be used in several contexts. It could be used as a mental model that guides the practice of emergency care for novice practitioners or it could be used as a teaching tool for medical students and trainees, in not only emergency care, but also other specialties that may have exposure to emergency cases. In addition to novice providers, it has implications for physicians in emergency departments, paramedics in emergency medical services, general practitioners in rural clinics, nurse practitioners, or anyone else practicing emergency care. This may lead to the development of training and educational methods that suit each stage separately, as well as recognizing cognitive biases and avoiding them.

The model may also be used for audits and reviews of emergency case management, including self-audits, departmental or institutional audits, or peer reviews. Moreover, clinical decision-making aids could be further developed and tailored to the needs of the practice. For example, algorithms and pattern recognition are suitable for steps B and C teaching and decision-making, while event-driven and hypothetico-deductive approaches are more suitable for step D. This model is very broad-based. It is hoped that this conceptual model will help practitioners develop a more focused approach, a broader perspective, and a better ability to detect critical signals when managing undifferentiated emergency cases.

## Data Availability

Yes
